# Healthcare disparities, screening, and molecular testing in the changing landscape of non–small cell lung cancer in the United States: a review

**DOI:** 10.1007/s10555-024-10187-6

**Published:** 2024-05-16

**Authors:** Razelle Kurzrock, Aadel A. Chaudhuri, David Feller-Kopman, Narjust Florez, Jed Gorden, Ignacio I. Wistuba

**Affiliations:** 1 Medical College of Wisconsin Cancer Center, Froedtert and Medical College of Wisconsin, 8701 Watertown Plank Rd, Milwaukee, WI 53226 USA; 2 Department of Radiation Oncology, Washington University School of Medicine, 4511 Forest Park Ave, St. Louis, MO 63108 USA; 3 Mayo Clinic, 200 First Street SW, Rochester, MN 55905 USA; 4 Geisel School of Medicine, Dartmouth Hitchcock Medical Center, One Medical Center Drive, Lebanon, NH 03756 USA; 5 Dana-Farber Cancer Institute, Harvard Medical School, 450 Brookline Ave - DA1230, Boston, MA 02215 USA; 6 Department of Thoracic Surgery and Interventional Pulmonology, Swedish Cancer Institute, 1101 Madison St, Suite 900, Seattle, WA 98104 USA; 7 Department of Translational Molecular Pathology, The University of Texas MD Anderson Cancer Center, 1515 Holcombe Blvd, Houston, TX 77030 USA

**Keywords:** Non–small cell lung cancer, Healthcare disparities, Lung cancer screening, Biomarker testing

## Abstract

Inequitable access to care continues to hinder improvements in diagnosis and treatment of lung cancer. This review describes healthcare disparities in the changing landscape of non–small cell lung cancer (NSCLC) in the United States, focusing on racial, ethnic, sex-based, and socioeconomic trends. Furthermore, strategies to address disparities, overcome challenges, and improve patient outcomes are proposed. Barriers exist across lung cancer screening, diagnosis, and treatment regimens, varying by sex, age, race and ethnicity, geography, and socioeconomic status. Incidence and mortality rates of lung cancer are higher among Black men than White men, and incidences in young women are substantially greater than in young men. Disparities may be attributed to geographic differences in screening access, with correlating higher incidence and mortality rates in rural versus urban areas. Lower socioeconomic status is also linked to lower survival rates. Several strategies could help reduce disparities and improve outcomes. Current guidelines could improve screening eligibility by incorporating sex, race, and socioeconomic status variables. Patient and clinician education on screening guidelines and patient-level barriers to care are key, and biomarker testing is critical since ~ 70% of patients with NSCLC have an actionable biomarker. Timely diagnosis, staging, and comprehensive biomarker testing, including cell-free DNA liquid biopsy, may provide valuable treatment guidance for patients with NSCLC. Efforts to improve lung cancer screening and biomarker testing access, decrease bias, and improve education about screening and testing are needed to reduce healthcare disparities in NSCLC.

## Introduction

Lung cancer has the second highest cancer incidence rate and is the leading cause of cancer-related mortality in the United States, with an estimated 238,340 new cases and 127,070 deaths projected for 2023 [1]. Non–small cell lung cancer (NSCLC) and small cell lung cancer are two primary lung cancer types that account for 81% and 14% of lung cancer cases, respectively [2]. Adenocarcinoma and squamous cell carcinoma are the most frequent histological types of NSCLC [2]. Most lung and bronchus cancer cases in the United States had a relative 5-year survival rate of only 23% in 2012–2018 [1]. However, in recent years, advances in immunotherapy and targeted therapies have improved disease prognosis and survival among patients with NSCLC and treatment strategies continue to shift towards targeted and small molecule therapies [3]. Mortality rates among patients with NSCLC rapidly declined shortly after recommendations for routine molecular testing and US Food and Drug Administration (FDA)–approved targeted therapies were introduced in 2013, with incidence-based mortality in men decreasing by 3.2% annually from 2006 to 2013 and then by 6.3% annually from 2013 to 2016; among women, incidence-based mortality decreased by 2.3% annually from 2006 to 2014 and then by 5.9% from 2014 to 2016 [4].

Lung cancer risk factors differ between men and women and include factors such as tobacco consumption, history of lung disease, genetic predisposition, and environmental or occupational exposures [2]. Lung and bronchus cancer incidence and mortality rates have steadily declined over the past two decades [1, 5], which may be attributed to the reduction in smoking rates; improved lung cancer screening, detection, and staging; and the development of new therapies [1]. However, barriers still exist across diagnostic and treatment pathways, varying by demographics including age, sex, race and ethnicity, geography, and socioeconomic status.

This review describes the changing landscape of NSCLC, focusing on racial and ethnic, sex-based, and socioeconomic trends and health disparities. We aim to increase awareness of barriers to equitable access to screening, diagnosis, biomarker testing, and treatment of NSCLC and highlight the importance of access to such care. Finally, we propose strategies to address disparities, overcome challenges, and improve patient outcomes.

## Discussion/observations

### Lung cancer patient population and healthcare disparities

#### Smoking status

Smoking is the leading cause of lung cancer, contributing to 82% of cases and 81% of deaths in the United States [2]. Increased smoking prevalence is mirrored by an increase in lung cancer incidence and mortality after a couple of decades. Lung cancer incidence and mortality rates have historically been higher among men versus women because the uptake of smoking in women occurred later than for men; however, women were also slower to quit smoking, which narrowed the gap in smoking rates between men and women [2]. Since the link between smoking and lung cancer was established, people have been motivated to stop smoking and the continued reduction in smoking rates is reflected by decreased lung cancer incidence by 2.6% and 1.1% per year among men and women, respectively, since 2006 [2]. In addition, the introduction of national screening guidelines in the early 2010s and subsequent expansion of lung cancer screening eligibility criteria have reduced lung cancer mortality rates by leading to earlier detection and treatment, particularly for high-risk populations: individuals aged 50–80 years with a 20-pack-year history, and individuals who stopped smoking within the last 15 years [6, 7].

**Fig. 1:**
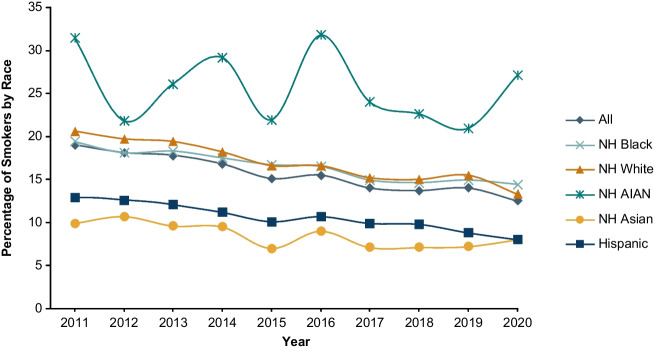
Percentage of US adult smokers by race from 2011 to 2020 [9]. *AIAN* American Indian or Alaska Native, *NH* non-Hispanic, *US* United States

When analyzed by race and ethnicity, smoking prevalence was historically greater among Black than White men, but since 1990, smoking rates among Black men have decreased to be similar to those of White men [2, 8]. Similarly, smoking prevalence has decreased among other ethnicities, with rates decreasing from 12.9 to 8.0% among Hispanic adults, 9.9 to 8.0% among non-Hispanic Asian adults, and 31.5 to 27.1% among non-Hispanic American Indian and Alaska Native (AIAN) adults from 2011 to 2020 (Fig. 1) [9]. Differences in smoking status across sex, race, and ethnicity may also be more evident among lung cancer cases with a history of low or moderate smoking levels [10, 11]. Additionally, smoking-related disparities may partly be driven by barriers to tobacco-cessation programs, poverty, and social conditions, as well as targeted marketing and advertising by the tobacco industry towards specific ethnic groups [9]. For example, the use of mentholated cigarettes has been reported in approximately 25% White, 30% Asian, 38% Hispanic, and 88% Black populations [12]. Such differences in smoking behavior impact inhalation patterns, nicotine dependence, and smoking cessation, consequently contributing to disparities in NSCLC incidence and mortality rates [13]. In October 2023, the FDA announced a proposed ban on the sale of menthol cigarettes and flavored cigars that could promote health equity in the United States and contribute to the reduction of race-related NSCLC disparities; however, the ban is yet to be implemented nationwide [14]. Even as the overall decrease in lung cancer incidence parallels a decrease in smoking prevalence, cases of NSCLC in nonsmoking individuals have been slowly increasing [15], particularly among women, individuals of a higher socioeconomic status, and individuals of Asian or Hispanic descent [15, 16].

#### Sex, race and ethnicity, and age

Lung cancer incidence and mortality rates vary by sex and race (Table 1) [2, 17]. Incidence and mortality rates are higher among White, Black, and AIAN men compared with Asian American, Pacific Islander (AAPI), and Hispanic men, with higher rates reported in Black than White men [2, 17]. Among women, the highest incidence and mortality rates have been reported among AIAN and White women [2]. The 5-year survival rate for lung cancer also varies by race and ethnicity, with AIAN patients experiencing the lowest survival rates, and White men having a higher survival rate than Black men [2].

**Table 1:** Lung cancer incidence rates,^a,b^ mortality rates,^a,b^ and 5-year survival rates^c^ in the United States by sex, race, and ethnicity [2]

Lung cancer rates	All	Black	White	AIAN	AAPI	Hispanic
**Incidence rate (2015–2019), cases per 100,000**						
Men	64.1	74.8	67.3	66.9^d^	42.1	35.6
Women	50.3	46.9	55.5	57.9	28.3	24.4
**Mortality rate (2016–2020), deaths per 100,000**						
Men	42.2	51.0	44.7	51.1^d^	25.6	20.9
Women	29.3	27.8	32.8	36.0	15.4	11.4
**5-year relative survival rate (2012–2018), %**						
Men	19	17	19	15	22	18
Women	27	25	27	24	31	28
Sexes combined	23	21	23	19	26	23

*AAPI* Asian American and Pacific Islander individuals, *AIAN* American Indian and Alaska Native individuals, *US* United States

^a^For AIAN individuals, incidence data are restricted to Purchased/Referred Care Delivery Area counties; mortality data are adjusted for misclassification on death certificates

^b^Age was adjusted to the 2000 US standard population

^c^Survival rates are for patients diagnosed during 2012–2018 and were followed through 2019. All racial groups exclude individuals identifying as Hispanic

^d^The mortality rate for AIAN men is disproportionate to the incidence rate

The prevalence of actionable biomarkers also varies with ethnic ancestry, further contributing to lung cancer disparities [18]. The frequency of somatic *EGFR* mutations is known to be higher among patients of East Asian descent (~ 45%) compared with those of European or African descent (~ 10%) and specific driver mutations in *EGFR*, *KRAS*, and *STK11* have been associated with Native American ancestry [18]. The high frequency of *EGFR* mutations observed in AAPI individuals may also underline the higher survival rate among this population compared with other race and ethnicities (Table 1), since patients with *EGFR*-positive NSCLC can be treated with *EGFR*-targeted therapeutic products [19]. Importantly, the frequency variation of these mutations in lung cancer is independent of smoking-related mutational processes and may contribute to the elevated risk of lung cancer in non-White never-smokers [18, 20].

Recent research also suggests that differences in incidence and mortality may partially be driven by racial and ethnic differences in the metabolism of carcinogens present in tobacco products, although access to care may also play a role [8, 10]. Tobacco carcinogens such as 4-(methylnitrosamino)-1-(3-pyridyl)-1-butanone, polycyclic aromatic hydrocarbons, 1,3-butadiene, and benzene have been found at higher levels in Black smokers than White smokers [10].

Despite societal beliefs that women are at lower risk of lung cancer than men, it is essential to recognize that lung cancer remains the leading cause of cancer-related death among women [1]. The age-adjusted incidence of lung cancer cases in the United States from 2015–2019 was 64.1 and 50.3 per 100,000 among men and women, respectively [2]. Per 2016–2020 mortality data, the number of deaths per 100,000 people was 42.2 for men and 29.3 for women [2].

**Fig. 2:**
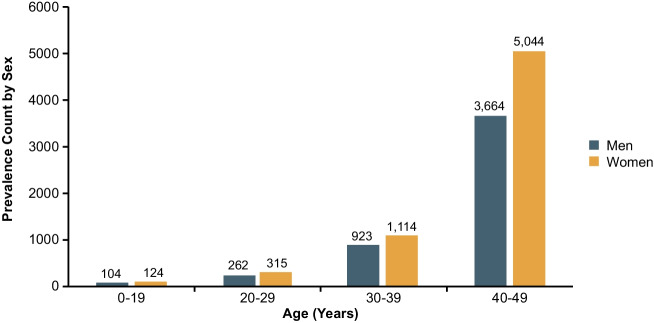
Lung cancer incidence among younger patients. Sex-specific 5-year limited-duration prevalence^a^ by age (0–49 years) for lung and bronchus cancer in the United States [23]. ^a^The estimated number of patients alive on a specific day (January 1, 2020) who were diagnosed with lung/bronchial cancer in the past 5 years (all races)

Age-related differences in lung cancer incidence and mortality have also been reported. Although most patients diagnosed with lung cancer are ≥ 55 years old, about 1% of newly diagnosed patients are < 45 years old, and lung cancer incidence in young women (30–49 years of age) has become substantially higher than in young men (Fig. 2) [5, 21–23]. Furthermore, between 2012 and 2018, patients aged 15–39 years had a 5-year relative survival rate of 50% [24]. Because of the relative rarity of lung cancer in young patients, routine lung cancer screening is only recommended for individuals ≥ 50 years old with a history of smoking [7]. This lack of routine screening in younger patients may explain why NSCLC is often diagnosed at advanced stages in young patients and may contribute to the high mortality rate in this population [25].

Young patients with lung cancer also tend to have a greater number of oncogenic genomic alterations, including *ALK* and *ROS* rearrangements and *ERBB2* (*HER2*) alterations (i.e., gene amplification or mutation), suggesting that this population may benefit from targeted therapy [21]. Other common mutations such as *BRAF* and *KRAS* appear to be associated with older populations; *EGFR* mutation prevalence, also present in young patients, seems to vary across studies [21]. Young women are also predominantly diagnosed with adenocarcinoma NSCLC and usually have low comorbidity scores [21, 26].

#### Geography and socioeconomic status

Lung cancer incidence and mortality are influenced by geography; Kentucky, West Virginia, Mississippi, and Arkansas continue to have the highest mortality rates throughout the United States [2]. These high mortality rates may be the result of rural populations having higher smoking rates (27.3% versus 17.7% in urban areas) and lower screening rates because of lack of access [27, 28]. Approximately 90% of individuals in isolated rural areas do not have access to screening facilities within 10 mi, which is more than 7 times higher in comparison with urban areas, where approximately 12% of individuals do not have access [28]. In addition, higher socioeconomic status is associated with greater 5-year survival rates compared with lower socioeconomic status [29]. Individuals from rural areas are more likely to be unemployed compared with individuals from urban areas (36% versus 28%) and median household income is also lower in rural areas compared with urban areas ($51,408 versus $68,388) [30]. The higher mortality rate among individuals having low socioeconomic status may be partly due to lower screening rates in this population [31].

### Lung cancer screening and diagnosis

#### Screening

As demonstrated in the National Lung Screening Trial (NLST), low-dose computed tomography (LDCT) screening reduced cancer-related mortality by an estimated 20% compared with chest radiography [6, 32]. Furthermore, results of the NELSON trial showed that after 10 years of follow-up, screening reduced mortality by 24% in men and 33% in women compared with no screening [33]. Consequently, the US Preventive Services Task Force (USPSTF) updated recommendations for high-risk patients in 2021, recommending annual LDCT screening in adults aged 50–80 years who have a 20 pack per year smoking history and currently smoke or have quit within the past 15 years [7]. The NCCN Clinical Practice Guidelines in Oncology (NCCN Guidelines^®^) for Lung Cancer Screening also recommend for individuals at high risk for lung cancer but with a negative LDCT scan or those whose nodules do not meet the size cutoff for more frequent scanning or other intervention to undergo annual LDCT screening until individuals are no longer candidates for definitive treatment [34]. However, only about 7% of eligible patients undergo LDCT screening in the United States [35].

#### Barriers and disparities

**Fig. 3:**
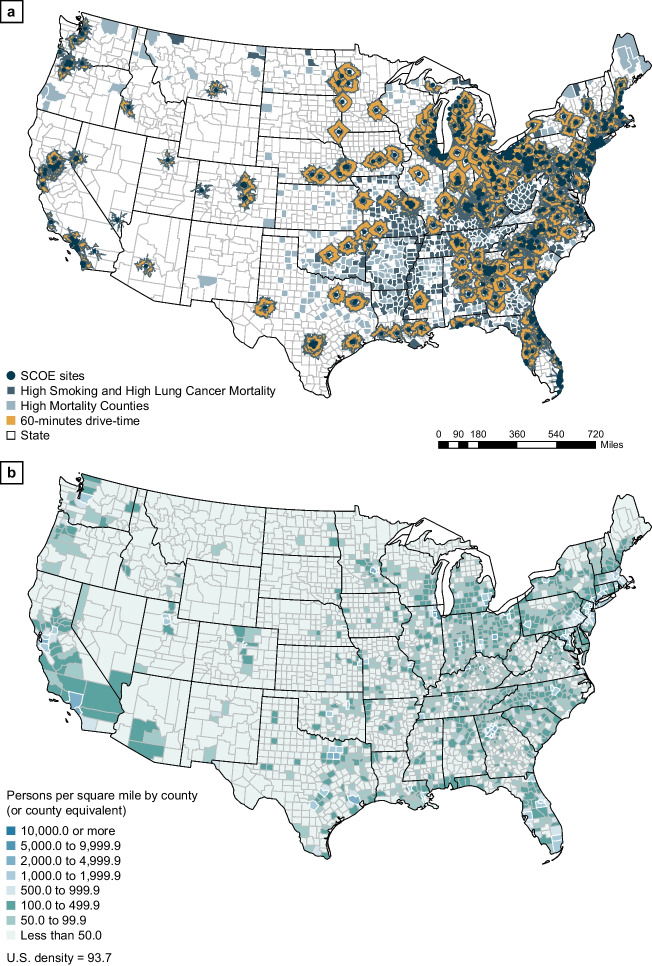
Distribution of lung cancer screening centers in rural and urban areas of the United States. **a** Distribution of SCOEs and counties with high smoking and lung cancer mortality rates beyond a 60-min drive from an SCOE [36]. Reprinted with permission of the American Thoracic Society. Copyright © 2023 American Thoracic Society. All rights reserved. S.J. Niranjan, W. Opoku-Agyeman, N.W. Carroll, A. Dorsey, M. Tipre, M.L. Baskin, and M.T. Dransfield. 2021. Distribution and geographic accessibility of lung cancer screening centers in the United States. *Annals of the American Thoracic Society.* 18(9):1577–1580. *Annals of the American Thoracic Society* is an official journal of the American Thoracic Society. **b** Urban and rural counties in the United States (persons per square mile by county; 2020 census demographic data) [39]. *SCOE* screening center of excellence

Several barriers limit the use of LDCT screening, including geographic location, racial and socioeconomic background, smoking status, age, and sex. Access to screening facilities varies by geographic location and across rural and urban environments [28, 35]. Although LDCT screening uptake is similar between rural and urban populations (16.3% vs 17.7%), in the United States, recognized screening centers of excellence providing high-quality LDCT screening are largely clustered in the more urban, Northeast and Midwest regions of the country (Fig. 3) [36, 37]. More than a third (36%) of counties with high mortality rates are at least a 60-min drive from an LDCT screening facility [36], illustrating that the inaccessibility of rural screening facilities may contribute to the higher incidence and mortality rates among rural populations. Geographic differences in screening access may also be attributed to the higher density of physicians per capita in urban areas, insurance rates, and socioeconomic status [38].

Individuals with a lower socioeconomic status, especially those with low educational levels, may face compounding challenges regarding lung cancer screening. These include limited knowledge and low perceived benefits of screening; fear of cancer diagnosis; fatalistic beliefs; and financial concerns regarding transportation, screening, and examination costs because they are more likely to be uninsured [31, 40]. More than half of patients who are eligible for lung cancer screening are uninsured or covered by Medicaid, which may not cover LDCT screening [38]. It should be noted that although the United States Centers for Medicare & Medicaid Services (CMS) provides coverage for annual lung cancer screening according to USPSTF eligibility criteria, this coverage is not necessarily for Medicaid beneficiaries since Medicaid eligibility is determined by the state [8].

Race and ethnicity may act as additional barriers to screening. Language barriers and the lack of awareness of lung cancer screening services have been reported in both Hispanic/Latinx and Asian populations [40]. In addition, the Hispanic/Latinx population has been reported to have a culturally rooted fear of a cancer diagnosis, and it is a cultural practice by Asian individuals to only seek medical care when ill; both of these cultural traits may play a role in individuals not undergoing screening [40]. Black individuals also face multilevel barriers to screening, including socioeconomic factors such as insurance status, financial constraints, and health literacy, as well as a historical mistrust of the healthcare system [8, 40, 41]. Indeed, eligible non-Black individuals are 2.8 times more likely to have had lung cancer screening than eligible Black individuals [42]. Further, smoking patterns among Black individuals may impact screening eligibility, because Black individuals have been shown to smoke fewer pack-years and are diagnosed at an earlier age [8, 43]. Even as updates to the USPSTF screening guidelines have been implemented to reduce these disparities, the criteria still favor older individuals with a longer smoking history, and screening criteria continue to be generated from clinical trials with majority White male populations [7, 44]. Regardless of race and smoking status, individuals aged < 50 years do not meet current screening criteria and are therefore less likely to receive a timely diagnosis [7]. Moreover, nonsmoking-related risk factors, such as secondhand smoke, occupational exposures (e.g., aerosolized cooking oils, radon exposure), and certain health conditions (e.g., prior malignancy, underlying lung disease), are not accounted for in current screening guidelines; this poses a challenge given the increasing proportion of nonsmokers diagnosed with NSCLC in recent years [15, 38].

These risk factors also disproportionately affect women more than men [45]; this, together with the observation that physicians are less likely to discuss lung cancer screening with women, places sex as an additional barrier to LDCT screening [46, 47]. To ensure equitable application of LDCT screening and minimize delays in diagnosis, it is imperative that guidelines continue to be refined to account for personalized risk, as well as sex and racial differences.

### Strategies to reduce lung cancer health disparities

#### Improving lung cancer screening rates

To reduce disparities in lung cancer screening, USPSTF screening criteria were expanded in 2015, causing a relative increase in eligibility by 30.3% for men, 31.9% for White populations, 40.5% for women, and 76.7% for Black, and 78.1% for Hispanic populations [48]. However, more information on the risks and benefits of lung cancer screening in diverse populations is needed to further tailor screening criteria [49].

Lower rates of follow-up after detection of lung nodules during lung cancer screening have been observed in Black and Latino/a patients, patients with a lower income status, and patients with mental health disorders [50]. The CMS mandates that screening requires a shared decision-making discussion with a healthcare professional (HCP); however, the currently available decision aids to be used in such discussions may not be appropriate for all racial and ethnic populations [49, 51]. The American Thoracic Society recommends that shared decision-making tools that are culturally sensitive and understandable across educational levels be developed and tested to address barriers that may affect treatment [49].

Healthcare institutions should also provide training on communication techniques during these discussions to help improve patient trust [49]. In addition, to reduce racial disparities in lung cancer screening, culturally adapted lung cancer screening marketing outreach programs, and partnerships between community screening sites and public health departments, medical societies, advocacy organizations, and patient navigators should be implemented to address patient concerns [49–51]. These programs and partnerships should be codeveloped with the target communities and focus on raising awareness not only in the marginalized subpopulations but also for HCPs [51].

Because physician referral plays an important role in LDCT screening, it is critical that HCPs (e.g., family physicians, oncologists, pulmonologists) receive appropriate education and training on modern screening guidelines and patient-level barriers to screening. This may help reduce implicit biases (e.g., racial, sex, socioeconomic status) that account for the lower physician referral rates among Black populations [38, 40].

Educating the public will also be paramount in improving screening rates. Relatable educational materials (e.g., videos, podcasts, patient testimonials) available in different languages and at appropriate reading levels should be readily accessible to the general population to convey the importance of lung cancer screening and promote screening in high-risk populations [49, 51]. Public awareness of and access to tobacco cessation programs may also improve screening rates in current smokers and should be developed with differences in literacy, language, and cultural beliefs in mind [49]; incorporating cessation programs into screening visits may improve smoking quit rates and improve outcomes for current smokers [8, 52].

Other approaches to address screening access include expanding telehealth coverage and dispatching mobile screening units to increase screening engagement in rural communities [38, 47, 49]. Ameliorating financial barriers to screening may also help improve uptake. Although several studies have shown that LDCT screening procedures are cost-effective and are on par with costs associated with other routine cancer screenings [53–55], in the real-world setting, the costs of LDCT screening often vary based on patient selection, false-positive test results and the associated additional workup, and any invasive procedures that need to be performed (e.g., needle biopsy, thoracotomy, thoracoscopy, and bronchoscopy) [7, 55]. Therefore, cost transparency from HCPs and financial assistance programs and care coordination for patients may help minimize financial constraints for the uninsured and those of lower socioeconomic status, potentially improving screening uptake [40]. In addition, institutions, HCPs, and advocacy groups should mandate Medicaid coverage for lung cancer screening nationwide [49].

#### Improving lung cancer diagnosis

The public perception that lung cancer is mainly associated with smoking obscures the message that lung cancer also occurs in individuals who have never smoked. Improving public awareness of nonsmoking-related lung cancer risk factors (e.g., environmental exposures, prior malignancy, secondhand smoke) and symptoms may encourage more patients to seek medical attention sooner [56].

Moreover*,* HCPs should be trained to recognize symptoms of disease, both in high-risk populations and in nonsmokers, to improve screening referral rates and diagnosis. Patients with prolonged or unexplained symptoms such as shortness of breath, a persistent cough, or chest pain that is unresponsive to treatment (for alternative diagnoses) should be referred for screening [57]. HCP awareness of these symptoms is especially important in nonsmokers because it has been shown that physician “detection bias” may delay their diagnosis [58]. The implementation of pulmonary nodule clinics with multidisciplinary teams may further improve the diagnostic accuracy and staging of patients' disease and reduce the time to treatment initiation [59].

#### Diversity in clinical trials

Demographic representation in clinical research is essential for assessing the efficacy and safety of novel therapeutic products in an equitable manner. However, some racial and ethnic groups are often under-represented in clinical trials [60]. Black individuals comprised only 4% of participants in the NLST trial on which USPSTF guidelines were based [44]; further, Black individuals form a mere 5% of national clinical trial enrollment but are 13% of the total United States population [41]. In a retrospective cross-sectional study on FDA-approved drugs for oncological conditions between January 2012 and December 2017, only 16% of the drugs were approved based on trials adequately representing Black patients, 20% representing Latinx patients, and 65% representing Asian patients [60]. The hesitancy of racial minority populations to participate in clinical trials may be due to a number of reasons. In the United States, being unable to speak and/or read English or being able to speak and/or read English at a certain level without full comprehension of what is said or written is a common barrier faced by individuals [61]. Other barriers include a lack of understanding about clinical trials, personal attitudes and beliefs, and a lack of trust in the healthcare system [41, 61, 62]. In addition, logistical barriers, such as the inability to access the healthcare or research center, also pose a problem for the recruitment and retention of minority populations in clinical trials [61]. Novel approaches will also be required to increase participation of underserved populations in biomarker-driven clinical trials [62]. Outreach programs within communities may be useful to bridge the knowledge and communication gaps in underserved communities [50, 51]. The language barrier may further be overcome by using bilingual staff, using material in non-English languages, and/or using an interpreter [61]. Patient-centered communication such as using simplified reading material and multimedia and/or social media may be another useful tool to bridge the communication gap [61]. Strategies that can be used to overcome the logistical barriers may include flexible timings and locations for study visits or home-based assessments [61]. Overall, the rates of inclusion in clinical trials should be monitored and pharmaceutical companies should be encouraged to set better diversity goals during trial recruitment [60].

### The importance of biomarker testing

Due to how molecular alterations have been linked to NSCLC, biomarker testing has dramatically improved cancer treatment decisions and patient outcomes in NSCLC [3, 63]. For example, patients with lung cancer who received matched targeted therapy based on biomarker testing results were shown to have a higher 5-year survival rate compared with those who received nontargeted therapy (83.8% vs 9.0%) [64]. About 69% of patients with advanced NSCLC may have potential actionable biomarkers and therefore be eligible for targeted treatments [65]. However, biomarker testing is not uniformly performed, often due to cost, lack of patient awareness, and lack of HCP expertise; an estimated 73% and 48% of academic and community clinicians, respectively, use biomarker testing for treatment decisions [66, 67]. It has also been reported that patients of low socioeconomic status and/or Black patients were less likely to undergo biomarker testing [41]. Furthermore, only 65–75% of patients with NSCLC and an actionable biomarker are treated with a targeted therapy (excluding immunotherapy) [68].

**Fig. 4:**
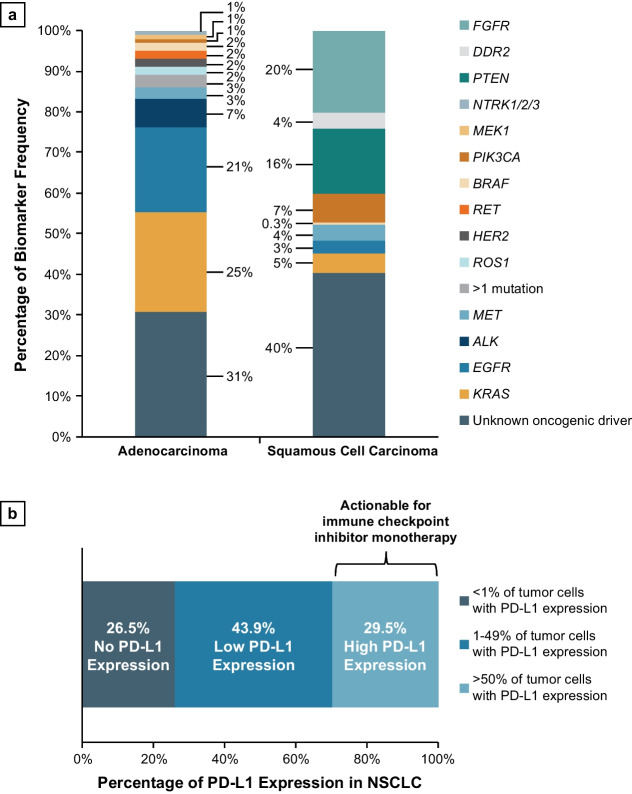
Frequency of current actionable molecular biomarkers in NSCLC. **a** Lung adenocarcinoma [65, 76] and squamous cell carcinoma biomarker frequency^a^ [71]. **b** PD-L1 expression in NSCLC tumor cells [73]. ^a^*ALK*, *ROS*, *NTKR*, *ERBB2* (*HER2*), and *RET* had listed frequencies of “none” for squamous cell carcinoma. *NSCLC* non-small cell lung cancer, *PD-L1* programmed cell death ligand 1

Both the American Society of Clinical Oncology (ASCO) and the NCCN Guidelines^®^ for Non–Small Cell Lung Cancer strongly advise broader molecular profiling, with the goal of identifying rare driver mutations for which effective drugs are available or to counsel patients on the availability of clinical trials [69, 70]. Adenocarcinoma and squamous cell carcinoma, the two most common NSCLC histological subtypes, are associated with unique biomarkers, which have varying frequencies depending on the type of NSCLC as depicted in Fig. 4a [65, 71]. Interestingly, *EGFR* and *KRAS* mutations appear to be more prevalent not only in adenocarcinoma, but also in Asian patients, women, and nonsmokers [65].

Microsatellite instability (MSI) and programmed cell death 1/programmed cell death ligand 1 (PD-1/PD-L1) are biomarkers associated with increased response by solid tumors to immunotherapy [72]. However, MSI is rare in NSCLC (reported in < 1% of patients with NSCLC), whereas PD-L1 expression is more common (approximately 30% of patients with NSCLC) [72, 73]. PD-L1 has a high predictive value of immunotherapy response rate (Fig. 4b); the higher the PD-L1 expression (i.e., PD-L1 expression in > 50% of tumor cells) the better chance the patient will respond to immunotherapy [72, 73]. High tumor mutational burden (TMB) is a tissue-agnostic biomarker for immune checkpoint inhibitor therapy with pembrolizumab, although it is not part of the NCCN Guidelines for NSCLC [74, 75].

Tissue next-generation sequencing (NGS) assays have shown promise in assessing biomarkers in patients because they allow simultaneous assessment of multiple biomarkers [77]. Tissue-based assays, such as FoundationOne CDx and Oncomine Dx Target Test, are approved by the FDA for the assessment of multiple biomarkers for NSCLC [78]. A well-known alternative to tissue genotyping is the sequencing of circulating cell-free tumor DNA (cfDNA) from a plasma liquid biopsy. Currently, the cobas EGFR Mutation Test v2, Agilent Resolution ctDx FIRST, FoundationOne Liquid CDx, and the Guardant360 CDx have been approved by the FDA for the assessment of cfDNA for NSCLC [78]. Interestingly, the Guardant360 assay, which has been analytically and clinically validated [79], demonstrated biomarker detection rates with plasma biopsies that are comparable to those in tissue in a prospective, multicenter cfDNA analysis in patients with previously untreated metastatic NSCLC [80, 81].

Because of the technologic advances of cfDNA NGS platforms and the increasing number of guideline-recommended biomarkers, the International Association for the Study of Lung Cancer (IASLC) recommends cfDNA, analyzed by a clinically validated NGS platform, as a valid tool for genotyping advanced NSCLC, and cfDNA should be considered superior to single-gene or polymerase chain reaction (PCR)-based approaches [77]. Although not guideline recommended, both PCR-based tests and liquid biopsy/cfDNA genotyping tests using NGS may be ordered during biomarker testing. Scenarios in which liquid biopsies/cfDNA genotyping are recommended as the initial approach for patients with advanced disease include patients with multiple mechanisms of acquired resistance to targeted therapies and when tissue samples are limited or inaccessible [77].

#### Collection and handling of tissue and/or blood samples for biomarker testing

In addition to good practices regarding tissue sampling, each institution should establish a formal molecular testing policy covering reflex testing, in which an NGS panel is automatically ordered for patients with preidentified histological types and stages [82]. Such an approach will not only help overcome disparities by ensuring that every patient receives the same comprehensive biomarker testing, but it has also been shown to improve turnaround times of results and improve detection rates [82, 83]. In regions where skilled personnel and laboratory resources are lacking, outsourcing of testing to independent laboratories or centralized regional testing centers may be the best approach to conserve resources and reduce costs through improved efficiency [74, 84]. Single-gene or low multiplexed-based approaches may also be valid options for clinicians in this setting [77].

#### Difficulties and possible solutions to comprehensive biomarker testing

Various clinical practice gaps for biomarker testing in advanced NSCLC can result in patients not receiving targeted treatment; attention to these gaps is needed to improve personalized care (Table 2). The constantly changing field of cancer genomics creates a challenge for HCPs and payers who must navigate the complex biomarker landscape; this can lead to knowledge gaps in financial options for patients, molecular testing strategies, and targeted therapy options [85, 86].

**Table 2:** Difficulties in biomarker testing and possible solutions [49, 61, 68, 77, 82, 87, 89, 90]

Difficulties in biomarker testing	Possible solutions
**Biopsies** • No biopsy conducted • Insufficient or overestimated tissue collection	**cfDNA liquid biopsy testing** • cfDNA samples can be useful when tissue samples are limited or inaccessible • cfDNA can also help assess patients with multiple mechanisms of acquired resistance to targeted therapies **Education** • Keeping up with rapidly evolving practice standards and treatments • Training programs, multidisciplinary tumor boards, and conferences • In-person and web-based education for practicing clinicians **Champion physician** • Nominating a local physician to educate local colleagues and healthcare teams on changing regulations **Nurse navigators** • Nurse navigators can help facilitate communication among multidisciplinary teams and support patient education **Patient advocacy organizations** • These organizations could help disseminate information to patients
**Biomarker test results** • No biomarker testing ordered • Inconclusive test results	
**Treatment** • Treatment initiated before biomarker testing was ordered or results received • Targeted therapy not selected after positive biomarker results	
**Reimbursement of NGS** • Sporadic/partial coverage by healthcare systems in the United States	**Reimbursement of NGS** • Knowledge of the revised 14-day rule by CMS in 2017 • Knowledge of the coverage determination listing issued by CMS in 2018 indicating that NGS as a diagnostic laboratory test is necessary and is covered nationally when specific requirements are met
**Clinical trials** • Participation of underserved populations in clinical trials	**Clinical trials** • Develop outreach programs within communities to bridge the knowledge and communication gaps on biomarker testing • Overcome the language barrier by using bilingual staff and/or an interpreter, by using simplified reading material published in non-English languages, and by using multimedia and/or social media • Include flexible timings and locations for study visits or home-based assessments to increase clinical trial recruitment and retention • Encourage pharmaceutical companies to set better diversity goals during trial recruitment

*cfDNA* cell-free deoxyribonucleic acid, *CMS* Centers for Medicare & Medicaid Services, *NGS* next-generation sequencing

HCP education on published guidelines*,* practical indications from clinical data, and financial resources for patients will assist HCPs in making informed decisions to guide treatment [85]. Furthermore, education about potential resource barriers in various healthcare environments may help HCPs appropriately manage biopsy samples for optimal molecular testing (e.g., prioritizing NGS testing over immunohistochemistry testing when tissue sample size is small). This knowledge can be gained not only through training programs, multidisciplinary tumor boards, and conferences but also through targeting practicing clinicians via in-person and web-based education [82, 86].

Practitioners should also consider selecting a local physician to partake in a “champion” role in which the physician becomes recognized as a lung cancer resource, educating local colleagues and healthcare teams on changing regulations [82]. Nurse navigators within the healthcare teams may also help with the molecular testing process and facilitate communication among multidisciplinary teams [82].

Reimbursement for NGS remains a barrier, despite being the most cost-effective approach to biomarker testing [87]. Mean cost of NGS testing has been estimated at $4932 per patient [67]; however, large healthcare systems in the United States provide only sporadic and/or partial coverage of these costs ($1269 to $2058 per test), with smaller healthcare systems providing hardly any coverage [87, 88]. Institutional, regional, and state-level changes may be required to overcome financial barriers to routine biomarker testing [86]. In 2017, the CMS revised the 14-day rule, which previously did not allow molecular diagnostic laboratories to bill CMS for tests ordered within 2 weeks after patient discharge; the revision updated the rule to accept certain advanced diagnostic and molecular pathology tests within 2 weeks of discharge [89]. In 2018, CMS also issued a coverage determination listing reporting that NGS as a diagnostic laboratory test is necessary and is covered nationally when ordered by a treating physician, performed in a Clinical Laboratory Improvement Amendments–certified laboratory, and when specific requirements are met [90].

## Limitations to disparities research and review

Challenges and limitations in disparities, screening, and molecular testing research and review include limited data on barriers to screening and access to healthcare facilities. In addition, research on healthcare literacy among minority populations and healthcare professional biases remain incomplete. Other areas that require additional research include costs and insurance coverage and their relationship to financial resources in various populations, as well as the methods of data collection and how they may affect disparities research. Moreover, infrastructure disparities, the effect of cultural and language barriers, and an understanding of the differences in access to and trust in technological advancement require a more in-depth exploration.

## Conclusions and future directions

Disparities in age, sex, geography, race and ethnicity, and socioeconomic status contribute to inequities in lung cancer screening access and utilization as well as to biomarker testing via genomic sequencing. There is ample value in timely diagnosis, staging, and comprehensive biomarker testing, including the use of liquid biopsy and cfDNA in addition to tissue biopsy, for treatment guidance in NSCLC and addressing access among underserved populations.

Addressing disparities in lung cancer care in the future requires a multifaceted approach that encompasses both research endeavors and healthcare policies. There should be an effort to have diverse representation in research studies, for instance, by restructuring clinical trial accrual goals and assessment methods. Additionally, efforts should be directed at regional and state-based changes for NGS reimbursement; developing community engagement and outreach programs; and improving equitable access to screening, biomarker testing, and treatment programs, including clinical trials. Addressing social determinants of health and promoting smoking cessation programs are crucial; health equity funding initiatives should be expanded. Finally, access to advanced precision medicine and personalized treatment approaches that consider the unique biological factors contributing to lung cancer across different populations will be a crucial part of addressing lung cancer disparities.

## Data Availability

Not applicable.
